# C-Terminal Extended Hexapeptides as Potent Inhibitors of the NS2B-NS3 Protease of the ZIKA Virus

**DOI:** 10.3389/fmed.2022.921060

**Published:** 2022-07-06

**Authors:** Suyash Pant, Nihar R. Jena

**Affiliations:** ^1^Department of Pharmacoinformatics, National Institute of Pharmaceutical Education and Research, Kolkata, India; ^2^Discipline of Natural Sciences, Indian Institute of Information Technology, Design and Manufacturing, Jabalpur, India

**Keywords:** Zika virus, NS2B–NS3 protease, peptide inhibitors, covalent inhibitors, MD-simulations, peptidomimetics

## Abstract

The Zika virus (ZIKV) protease is an attractive drug target for the design of novel inhibitors to control the ZIKV infection. As the protease substrate-binding site contains acidic residues, inhibitors with basic residues can be beneficial for the inhibition of protease activities. Molecular dynamics (MD) simulation and molecular mechanics with generalized Born and surface area solvation (MM/GBSA) techniques are employed herein to design potent peptide inhibitors and to understand the nature of the basic residues that can potentially stabilize the acidic residues of the protease substrate-binding site. It is found that the inclusion of K, R, and K at P1, P2, and P3 positions, respectively, and Y at the P4 position (YKRK) would generate a highly stable tetrapeptide-protease complex with a ΔG_bind_ of ~ −80 kcal/mol. We have also shown that the C-terminal extension of this and the second most stable tetrapeptide (YRRR) with small polar residues, such as S and T would generate even more stable hexapeptide-protease complexes. The modes of interactions of these inhibitors are discussed in detail, which are in agreement with earlier experimental studies. Thus, this study is expected to aid in the design of novel antiviral drugs against the ZIKV.

## Graphical Abstract

Binding of YKRKST to the protease, which fully occupies its substrate-binding site and hence may act as a potent inhibitor of the ZIKA virus protease. 
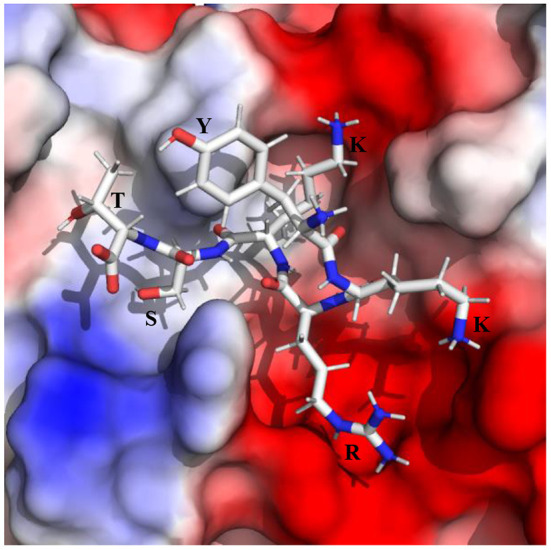

## Introduction

The Zika virus (ZIKV) infection causes both mild and severe diseases including fever, joint pain, (1, 2), Guillain-Barre syndrome (3), acute myelitis (4), and brainstem dysfunctions (5). In the case of infected pregnant women, it induces microcephaly (6), and other congenital malformations (7–9). Although ZIKV infection was declared a global emergency by the World health organization (WHO) in the year 2016 (10), no approved vaccine or drug is available to date to contain this disease (11–13).

The ZIKV contains a single-stranded RNA genome that translates to form a polypeptide chain inside host cells. Subsequently, this polypeptide gets cleaved by the host and viral proteases to form three structural [Envelop (E), membrane (M), and capsid (C)] and seven non-structural (NS1, NS2A, NS2B, NS3, NS4A, NS4B, NS5) proteins (14). Among these proteins, the NS3 encodes the serine protease, RNA helicase, RNA 5' triphosphatase (RTPase), and nucleocapsid triphosphatase (NTPase) activities (14–17). The N-terminal region of NS3 in association with the membrane-bound NS2B co-factor constitutes the serine protease (14, 15), which is responsible for the cleavage of the viral polyprotein and different key host proteins involved in immune response (18). Therefore, it is necessary to design substrate competitive inhibitors to inhibit the protease activities of NS2B–NS3 protease to contain the ZIKV (19–35).

Several attempts were made to design substrate competitive inhibitors to occupy the substrate-binding site of the NS2B–NS3 protease of the ZIKV. These inhibitors include (1) small molecules (19–27), (2) peptidomimetics (28–33), and (3) peptide inhibitors (34, 35). As the protease active site is surrounded by negatively charged amino acids (acidic residues), the binding of small molecule neutral inhibitors was not effective. However, peptidomimetic inhibitors that contain positively charged amino acids (basic residues), such as Lys (K) and Arg (R), and different organic warheads were proposed to be efficient (29, 30). These inhibitors can either make a covalent bond with the catalytically important residue Ser135 of NS3 (28, 29) or bind non-covalently to the protease (30). Interestingly, recently, non-covalent peptide inhibitors were shown to bind strongly with the NS2B–NS3 protease (35). Among these inhibitors, the YKKR was found to possess the highest binding free energy (35). It was shown that the P1 R binds to the S1 substrate site, while P2 K and P3 K bind to the S2 and S3 sites, respectively. It was proposed that the heavy amino acid Y at the P4 position provides the necessary conformational rigidity to KKR to fully occupy the substrate-binding site (35). However, for the Dengue virus (DENV) protease, strong preferences for both K and R were reported at the P1 position, whereas, R and K were preferred at the P2 and P3 positions, respectively (36). For the West Nile Virus (WNV), K/R at the P1 position and K at both P2 and P3 positions were found to be preferred (37, 38). As the DNV and WNV proteases are structurally similar to that of the ZIKV (39), it is necessary to understand the binding preferences of K and R at different substrate sites of the ZIKV protease. This will eventually help in the development of potent inhibitors for the inhibition of ZIKV protease activities.

Although the bindings of different inhibitors to the unprimed sites (S1–S4) are well studied (19–35), the inhibitor binding to the prime sites (S1' and S2') is rarely studied. Therefore, it is also desirable to understand the effects of prime site residues on the stability of peptide-protease complexes. Analysis of sequences of different flavivirus substrates indicates that Ser (S) and Thr (T) are present at the P1' and P2' positions respectively (36). As these polar residues are small in size, they can be well accommodated in the small cleft made by the S1' and S2' substrate sites of the protease. Therefore, the C-terminal extension of the most stable tetrapeptide inhibitors by S and T may further stabilize the peptide-protease complexes.

To identify a promiscuous inhibitor of the ZIKV protease, the positions of K and R in the YKKR-protease complex were changed without perturbing the P4 Y to generate 7 different peptides. Subsequently, the structural and dynamical effects of these inhibitors bound to the protease were undertaken to elucidate their roles in creating closed complex structures. Eventually, the relative Gibbs binding free energy analysis was carried out to short-list the most stable peptide-protease complex. The two most stable complexes were further extended by adding S and T to their C-terminal ends to understand the effects of prime residues on the inhibitor binding.

## Computational Methodology

### System Preparation

Recently, the binding of YKKR to the bZipro form of the protease (40) was shown to produce a stable complex with a relative binding free energy of about −73 ± 8 kcal/mol (35). The average simulated structure of the YKKR-protease complex was found to be similar to the X-ray structures of Acyl-KR-Aldehyde-protease (PDB ID 5H6V) (29), Phenylacetyl-KKR-protease (PDB ID 5ZMQ) (30), and TGKR-protease (PDB ID 5GJ4) (34) complexes. For this reason, the average simulated complex structure of the YKKR (35) was converted to seven different peptides, such as YKKK, YKRK, YRKK, YKRR, YRKR, YRRK, and YRRR by mutating P1 R, P2 K, and P3 K without perturbing the initial backbone conformation of YKKR. The PyMOL program (41) was used to create the mutated peptides. As these peptides were subjected to molecular dynamics simulations, it is believed that the mutated structures are not biased to their initial conformation (35, 42, 43).

### Molecular Dynamics (MD) Simulations and MM/GBSA Calculations

The Desmond 2021-1 program of Schrodinger (44, 45) was used to solvate the peptide-protein complexes by placing them in an explicit water box of size 10 Å. The OPLS4 force field (46) was used to model the peptide inhibitors. The partial charges of the ligands were generated by using the same force field. The single-point charge (SPC) model (47) was used to account for the explicit water molecules. Sufficient numbers of ions were added to make the solvated complexes neutral. The protonation states of the protein and peptidomimetic ligands were set as per the *pH* = 7.4. Subsequently, these complexes were energy minimized by 2,000 steps each by using the steepest descent and limited-memory Broyden-Fletcher-Goldfarb-Shanno (LBFGS) algorithms (48). The minimized complexes were slowly heated to maintain a temperature of 300 kelvin (K) in several steps by using the Nose-Hoover thermostatic algorithm (49). In the first step, the system was heated to 10 K for 100 ps. to reduce any possible steric clashes. In the second step, a 12 ps. of molecular dynamics run was performed with the NVT ensemble to relax the system at 10 K. In the third step, molecular dynamics run of 12 ps. was carried out by using the NPT ensemble, where a pressure of 1 atm was maintained by using the Langevin barostat (50). In the fourth step, the temperature was raised to 300 K for 12 ps. by using the NPT ensemble. In all of the above steps, the solute heavy atoms were restrained with a force constant of 50 kcal mol^−1^Å^−1^. In the fifth step, restraint was released and the molecular dynamics simulation was carried out at the NPT ensemble for 24 ps. Consequently, all complexes were subjected to a production run for 200 ns by considering the integration time step of 1 fs and the NPT ensemble. The periodic boundary condition (PBC) (51) was considered for all of the simulations.

To calculate the relative binding free energy (ΔG_bind_) of each protease-inhibitor complex, the MM/GBSA technique as implemented in the Desmond 2021-1 package (45, 46) was used. For this purpose, 100 snapshots were extracted from the last 10 ns trajectories of each complex at an interval of 100 ps. Equation (1) was used to compute ΔG_bind_.


(1) $$\begin{array}{l} G_{bind=G_{complex\;(minimized)}-G_{protein\;\left(unbound,minimized\right)}}\\ \;-G_{ligand\;(unbound,minimized)} \end{array}$$

where, G_complex(minimized)_ is the MM/GBSA energy of the minimized complex, G_protein(unbound, minimized)_ is the MM/GBSA energy of the minimized protein after separating it from its bound ligand and G_lig(unbound, minimized)_ is the MM/GBSA energy of the ligand after separating it from the complex and allowing it to relax. However, as entropy calculations were not performed, the free energy terms contain contributions from enthalpy only.

## Results and Discussions

**Figure 1:**
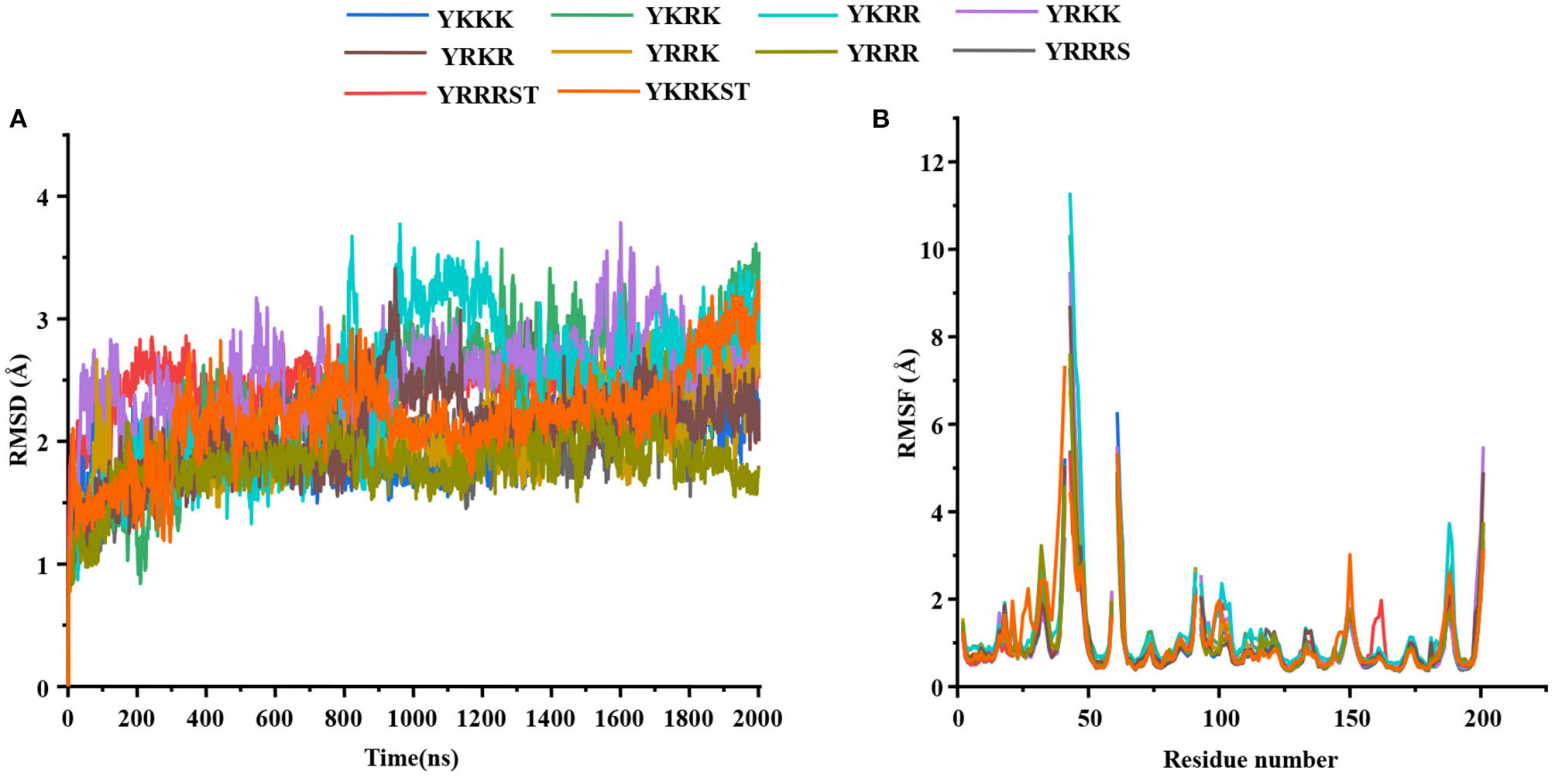
**(A)** The root mean square deviations (RMSD) (Å) of the C_α_ atoms of the protease and **(B)** the root mean square fluctuations (RMSF) (Å) of different residues of the protease in different protein-peptide complexes.

The root mean square deviations (RMSD) of the Cα atoms of the protease and the root mean square fluctuations of different residues of the protease evolved during the simulations are illustrated in Figure 1. As the average RMSD of each complex is <2.5 Å, the complexes were stable during the simulations. The interaction diagrams (including hydrogen bonding, electrostatic, π-cation, π-π interactions, etc.) depicted in Supplementary Figures S1–S10 elucidates the detailed interactions of the peptide inhibitors with the protease evolved during the simulations. The interactions that lasted for ≥80, 50–79, and <50% of the simulation time are considered to be strong, moderate, and weak, respectively (49). It should be mentioned that a hydrogen bonding interaction satisfies the following geometric criteria : (1) The protein-ligand H-bond distance is ≤ 2.5 Å between the donor and acceptor atoms (D–H···A), (2) a donor angle of ≥120° exists between the donor-hydrogen-acceptor atoms (D–H···A), and (3) an acceptor angle of ≥90° exists between the hydrogen-acceptor-bonded atoms (H···A–X). The π-cation interaction is defined as the interaction between an aromatic and charged groups situated within a distance of 4.5 Å. Similarly, the π-π interaction is defined as the interaction between two aromatic groups stacked face-to-face or face-to-edge by maintaining a distance of <4.5 Å. The relative binding free energies (ΔG_bind_) of different peptide-protease complexes presented in Table 1 indicate the binding strength of different peptide inhibitors studied herein.

**Table 1:** The relative binding free energies (ΔG_bind_) of different complexes.

**Complex**	**ΔG_BIND_ (kcal/mol)**
YKKR-protease	−72.88 ± 8.15^a^
YKKK-protease	−59.16 ± 6.69
YKRK-protease	−80.52 ± 6.84
YKRR-protease	−62.47 ± 8.25
YRKK-protease	−72.87 ± 7.25
YRKR-protease	−56.33 ± 7.92
YRRK-protease	−74.02 ± 9.08
YRRR-protease	−77.94 ± 6.90
YRRRS-protease	−84.84 ± 8.46
YRRRST-protease	−107.49 ± 10.40
YKRKST-protease	−96.84 ± 9.21

a *Ref. (35)*

### The Most Stable Peptide-Protease Complex

**Figure 2:**
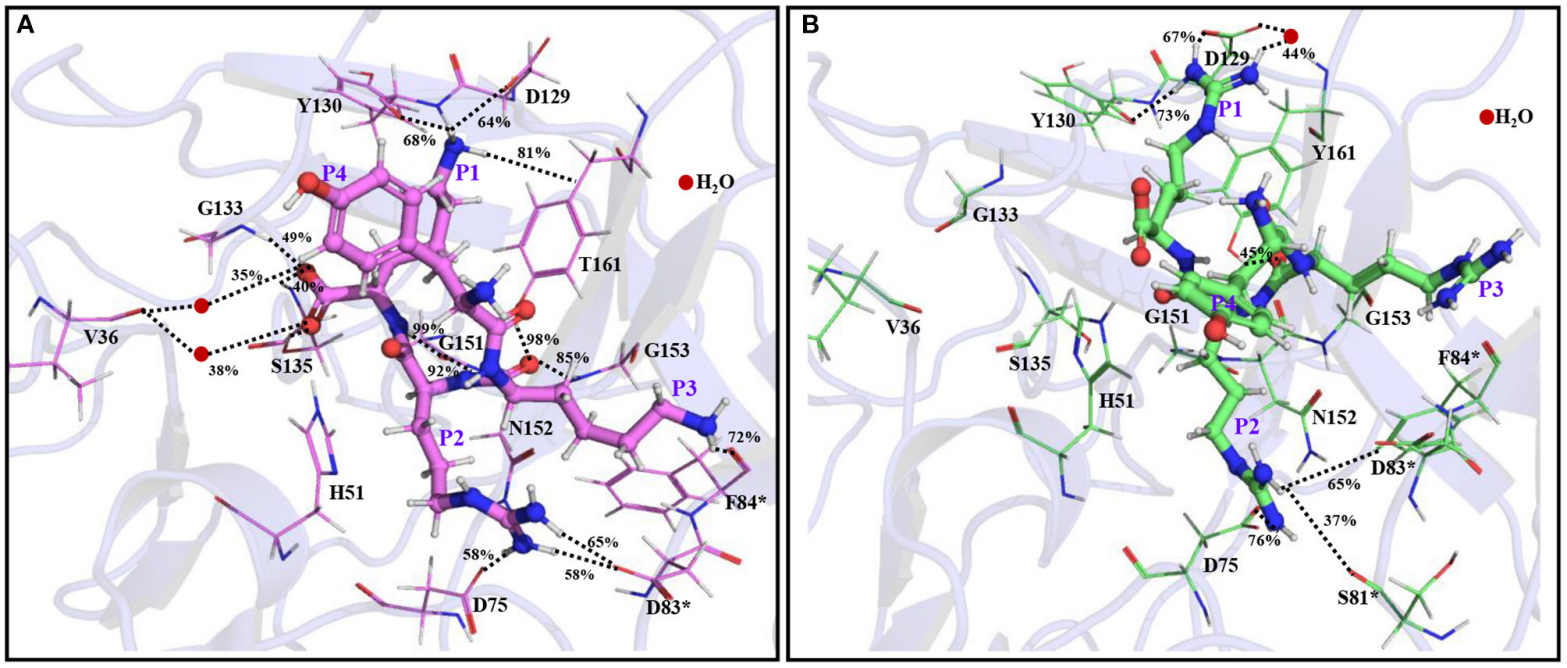
The average simulated structures of **(A)** YKRK-protease and **(B)** YRRR-protease complexes. The residues of NS2B are marked by *. The hydrogen bonding and ionic interactions are shown by dotted lines. The percentage occupations of different interactions are also mentioned. The P1-P4 residues of the inhibitors are labeled.

Most of the peptidomimetics that contain di- or tripeptides have R at the P1 position and K at the P2 position (28–31). We have also recently shown that YKKR tetrapeptide with R and K at the P1 and P2 positions, respectively binds strongly to the protease (35). However, the consideration of alternate combinations of R and K at P1, P2, and P3 positions revealed that the stabilities of different complexes involving tetrapeptides would follow the order YKRK > YRRR > YRRK >YRKK ≥ YKKR > YKRR >YKKK > YRKR (Table 1). This indicates that the YKRK-protease complex would be the most stable one, which is about 8 kcal/mol more stable than the YKKR-protease complex (Table 1). The YKRK peptide contains P1 K instead of P1 R, clamped by P2 R and P3 K. It is also about 3 kcal/mol more stable than the second most stable complex of this series (YRRR-protease). As both YKRK and YRRR contain P2 R and P4 Y and the P3 residue does not contribute to the acid-base interaction, the slightly higher stability of the former complex is likely due to the stronger binding of P1 K with the residues of S1 site. This is evident from Figure 2 and Supplementary Figure S1, where P1 K is more suitably placed in the S1 site to make a salt-bridge interaction with Asp129 (64% occupancy), a hydrogen bond with Tyr130 (68% occupancy), and a π-cation interaction with Tyr160 (81% occupancy) of NS3 (Figure 2A). Additionally, its backbone amide makes a strong hydrogen bond with Gly151 (92% occupancy) of NS3. However, in the case of YRRR, the positively charged guanidine group of P1 R points away from Asp129 and missed the key ionic interaction with Asp129 (Figure 2B). Further, the C-terminal carboxyl group of YKRK makes direct and indirect hydrogen bonds with S135, Gly133, and Val36 (<50% occupancy) of NS3 (Figure 2A), which are missing in YRRR (Figure 2B). Similarly, the side chain of P2 R in YKRK makes moderate salt-bridge interactions with Asp75 (58% occupancy) of NS3 and Asp83^*^ (58% occupancy) of NS2B and a moderate hydrogen bond with Asp83^*^ (65% occupancy) of NS2B. Its backbone also makes a strong hydrogen bond with Gly151 (99% occupancy) of NS3 (Figure 2A). We also noted that the P3 K of YKRK makes a moderate hydrogen bond with Phe84^*^ (72% occupancy) of NS2B and is favorably placed in the S3 site to facilitate long-range electrostatic interactions with Asp75 (NS3), Asp79^*^ (NS2B), and Asp83^*^ (NS2B). Although P2 R in YRRR can make a moderate salt-bridge interaction with Asp83^*^ (65% occupancy) of NS2B, it failed to make an ionic interaction with Asp75 of NS3 (Supplementary Figure S7A). However, it can make moderate and weak hydrogen bonds with Asp75 (76% occupancy) of NS3 and Ser81^*^ (37% occupancy) of NS2B, respectively (Figure 2B). Remarkably, the P3 R in YRRR failed to interact with the NS2B residues (Figure 2B; Supplementary Figure S7A). These results suggest that the binding of YRRR to the protease is weaker compared to YKRK.

Interestingly, if we compare the ΔG_bind_ of YKKR-protease and YRRR-protease complexes, it is clear that the latter complex is about 5 kcal/mol more stable than the former. This is in agreement with an earlier study (30) where a slightly higher IC_50_ value was obtained for a peptidomimetic inhibitor that contained RRR compared to KKR. It should be mentioned that as both YKKR and YRRR contain P1 R but differ at the P2 residue and the P3 residue does not make any direct ionic interaction with the protease, the higher stability of the latter is mainly due to the stronger binding of P2 R with the residues of S2 site. These results indicate that an ionic peptide with K at the P1 position and R at the P2 position may yield better potency. Also, the higher ΔG_bind_ of YKRK suggests that K would be preferred at the P3 position. In an earlier kinetic study for ZIKV (37), K was found to be preferred at the P3 position, while neutral residues, such as Trp, Tyr, Asp, and Pro at the same position showed no substrate activity. Similarly, in all stereotypes of the DENV protease, K/R at P1, R at P2, and K at P3 position were found to be highly favored (36). The yellow fever virus also prefers K/R, R, and K at P1, P2, and P3 positions, respectively (37). However, for the WNV protease, K/R at the P1, K at P2, and K at the P3 position were found to be preferred (37, 38).

It should be mentioned that the bindings of different pentapeptide substrates, such as (1) acyl-Norleucine-Lysine-Lysine-Arginine-7-amino-4-carbamoyl-methyl coumarin (Ac-nKKR-ACC), (2) acyl-D-Arginine-Lysine-Ornithine-Arginine-7-amino-4-carbamoyl-methyl coumarin (Ac-D-RKOR-ACC), (3) acyl-D-Lysine-Lysine-Ornithine-Arginine-7-amino-4-carbamoyl-methyl coumarin (Ac-D-KKOR-ACC), and (4) benzoyl-Norleucine-Lysine-Arginine-Arginine-aminomethyl coumarin (Bz-nKRR-AMC) to the ZIKV protease were shown to produce stable complexes of ΔG_bind_ lying between −20.42 ± 5.26 and −43.53 ± 0.97 kcal/mol (without entropy calculations) (52). Among these substrates, the Ac-D-RKOR-ACC-protease complex was found to be the most stable one (52). This result led the authors to propose that R and O are preferred at P1 and P2 positions, respectively. However, as the study was not undertaken by considering P1 K and P2 K, the results obtained therein are not conclusive (52). Further, as P1 R and P2 O in the Ac-D-RKOR (substrate 2) were not making key ionic interactions with Asp129 and Asp83^*^ of S1 and S2 sites, respectively, the Ac-D-RKOR-protease complex will be less stable than that of the YKRK-protease complex (52). Although the ΔG_bind_ values of the above substrate-protease complexes were obtained by using the MM/PBSA technique and the AMBER16 force field (52), the results cannot be directly correlated with the results obtained herein. However, the binding modes of the substrates/inhibitors and the relative difference in their ΔG_bind_ values support the higher stability of YKRK compared to Ac-D-RKOR.

### The C-Terminal Extension of YRRR

As the C-terminal carboxyl group of some of the tetrapeptides including YKRK makes indirect hydrogen bonds with residues of prime sites, the C-terminal extension of these peptides may likely generate additional interactions with the residues of S1' and S2' sites. For these reasons, the two most stable peptides (YKRK and YRRR) were extended by adding one or two residues at their C-terminal. Initially, the YRRR was extended by adding S to its C-terminal (P1' position). Subsequently, the YRRRS peptide was further extended by adding T at the P2' position. These extended peptides can behave as the substrate competitive inhibitors and fully occupy the binding site of the protease.

**Figure 3:**
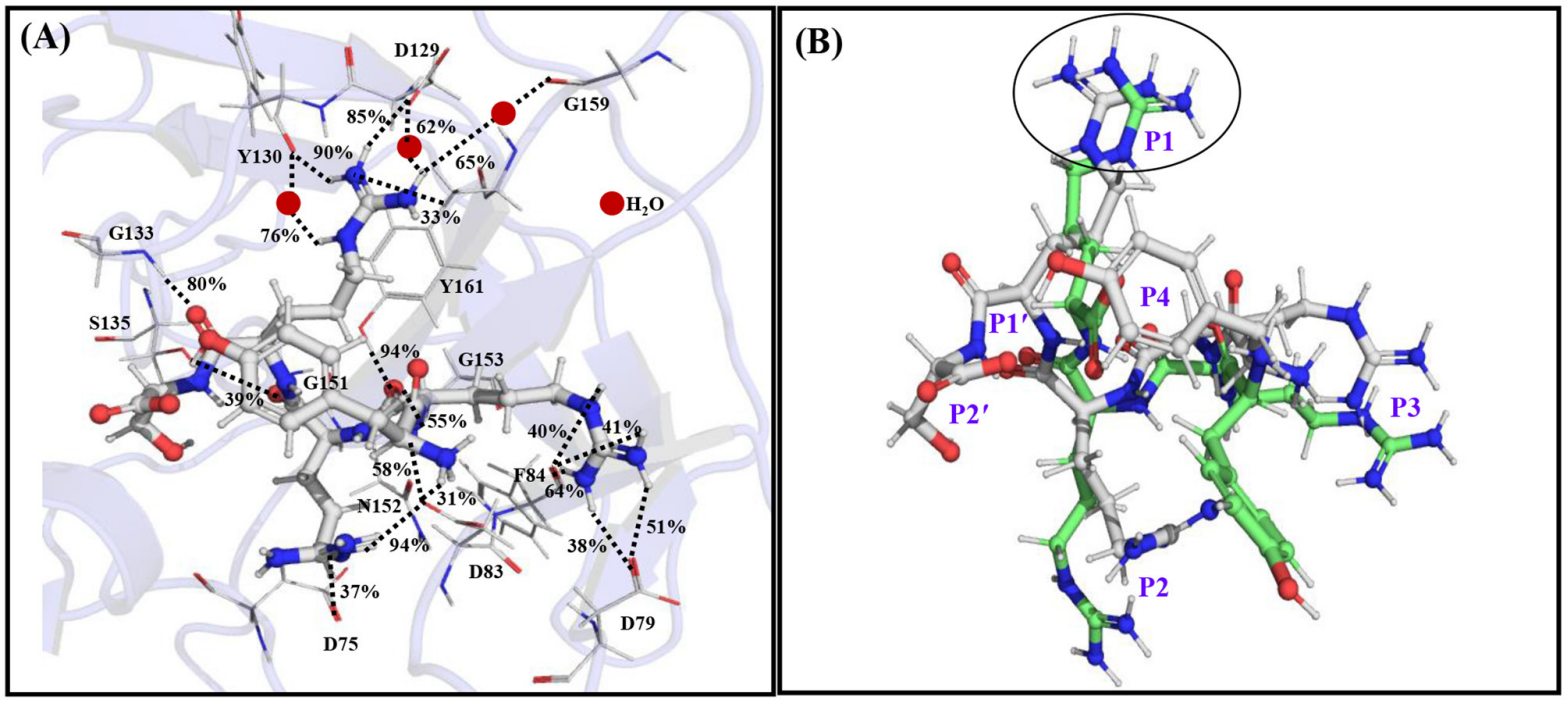
**(A)** The average simulated structure of the YRRRS-protease complex. The different interactions between the peptide and protease are shown by dotted lines and the percentage occupations of these interactions evolved throughout the simulation are also mentioned. **(B)** The superimposition of the average simulated structure of the YRRRS-protease [**(C)** atoms are shown in white] onto the average simulated structure of the YRRR-protease [**(C)** atoms are shown in green] complex (protease structures are not shown). The P2'-P4 residues of the inhibitors are labeled in **(B)**.

It is found that the YRRRS-protease complex is about 7 kcal/mol more stable than that of the YRRR-protease complex and the YRRRST-protease complex is about 23 kcal/mol more stable than the YRRRS-protease complex (Table 1). This indicates that the C-terminal extension of YRRR by P1' S and P2' T will enhance its stability by 30 kcal/mol. Interestingly, the positively charged guanidine group of P1 R in YRRRS rotated 180 degrees from its initial conformation (YRRR) to make a strong ionic interaction with the Asp129 (85% occupancy) (Figure 3B, Supplementary Figure S7). Further, in this conformation, it makes several direct and indirect interactions of moderate stability with Asp129, Tyr130, and Tyr161 of NS3 (Supplementary Figure S7B). The P2 R makes weak interactions with Asp75 of NS3 and Asp83^*^ of NS2B (<50% occupancy) and P3 R makes several interactions (<50% occupancy) with Asp79^*^ and Phe84^*^ of NS2B (Figure 3A, Supplementary Figure S7B). Notably, the ionic interaction between P3 R and Asp79^*^ of NS2B is missing in the YRRR-protease complex (Figure 2B). Moreover, the N-terminal Y moved up toward the C-terminal S and its terminal ${\text{NH}}_{3}^{+}$ group makes a weak electrostatic interaction with Asp83^*^ of NS2B (31% occupancy). Other than these, the backbones of P1' S, P2 R, P3 R, and P4 Y are found to make moderate hydrogen bonds with Gly133, Ser135, Gly153, Tyr161, and Asp83^*^ (Figure 3A). Although only the backbone interaction of P1' S with Gly133 (80% occupancy) is obtained, its inclusion at the C-terminal helped P1 R to make stronger interactions with the residues of the S1 site, in particular with Asp129. These results suggest that YRRRS would make stronger interactions with the protease compared to YRRR.

**Figure 4:**
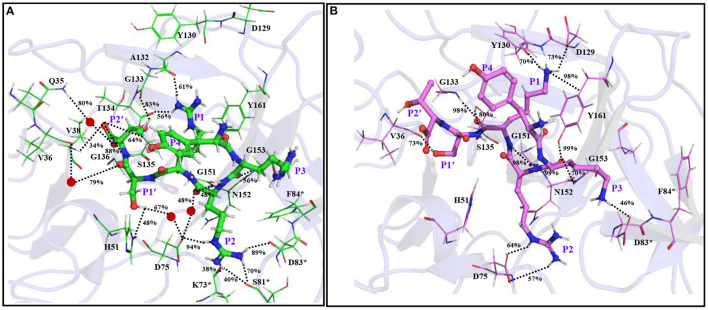
The average simulated structures of the **(A)** YRRRST-protease (carbon atoms in green) and **(B)** YRKRST-protease (carbon atoms in violet) complexes. Different interactions are shown by dotted lines and their percentage occupations evolved during the simulations are also mentioned. Residues of NS2B are marked by *. The P1'–P4 residues of the inhibitors are labeled.

Remarkably, further extension of YRRRS to YRRRST abolished interactions of P1 R with Asp129, Tyr130, and Y161 (Figure 4A; Supplementary Figure S9A) as the loop containing these residues moved away from the inhibitor. However, it made new contacts (hydrogen bonds) with Ala132 (61% occupancy) and Tyr134 (56% occupancy) and maintained the same orientation of the guanidine group as is obtained in the case of YRRRS (Figure 3A; Supplementary Figure S7B). In this structural arrangement, the side-chain hydroxyl group of P1' S makes a direct but weak hydrogen bond with His51 (48% occupancy) and a water-mediated moderate hydrogen bond with Asp75 (67% occupancy). Its carbonyl backbone also makes a water-mediated moderate hydrogen bond with Val36 (79% occupancy) (Figure 4A; Supplementary Figure S9A). The P2' T is found to make stable direct hydrogen bonds with Gly133 (83% occupancy) and Gly136 (88% occupancy) and a moderate hydrogen bond with Ser135 (64% occupancy). It also makes stable indirect water-mediated hydrogen bonds with Gln35 (80% occupancy). Interestingly, the interactions of P2 R with the residues of the S2 site improved significantly. It is now making a moderate ionic interaction with Asp75 of NS3 (74% occupancy) and strong hydrogen bonds with Asp83^*^ of NS2B (89% occupancy) and Asp75 of NS3 (94% occupancy). It also makes moderate and weak hydrogen bonds with Ser81^*^ (70% occupancy), and Lys73^*^ (38% occupancy) of NS2B, respectively (Figure 4A; Supplementary Figure S9A). Other than these, the backbone atoms of P2 R and P3 R are making direct and indirect hydrogen bonds with Asn152, Asp75, and Gly153 of NS2B. However, the side-chain of P3 R and P4Y do not make any contact with the protease. Hence, the tight binding of the inhibitor with the prime site residues may have contributed to the higher stability of the YRRRST-protease complex.

### The C-Terminal Extension of YKRK

As the binding of YRRRST hexapeptide to the protease is more stable than that of YRRRS, we added S and T to the C-terminal of YKRK to generate YKRKST-protease complex (Figure 4B; Supplementary Figure S9B). It is found that the YKRKST-protease complex is about 16 kcal/mol more stable than the YKRK-protease complex (Table 1). However, it is about 11 kcal/mol less stable than the YRRRST-protease complex (Table 1). This is a bit surprising and indicates that the preferences of K, R, and K at the P1, P2, and P3 positions, respectively get lost after the inclusion of P1' and P2' residues. Interestingly, a moderate hydrogen bond between the P1' S and Val36 (73% occupancy) is the only interaction found between the inhibitor and the prime site residues (Figure 4B; Supplementary Figure S9B). However, the P1 K was placed in the S1 site and is making a moderate electrostatic interaction with Asp129 (73% occupancy), a moderate hydrogen bond with Tyr130 (70% occupancy), and a strong π-cation interaction with Tyr161 (98% occupancy) (Figure 4B). Its carbonyl backbone is also making strong hydrogen bonds with Gly133 (98% occupancy) and Ser135 (83% occupancy) of NS3 and amide backbone is making a strong hydrogen bond with Gly151 (98% occupancy) of NS3. This clearly shows that the binding of P1 K to the S1 site remains to be strong despite the C-terminal extension. Although the side chain of P2 R makes an ionic and a hydrogen bond each with Asp75 (>50% occupancy) of NS3 it could not interact with Asp83^*^ of NS2B. Nevertheless, its amide backbone makes a strong hydrogen bond with Gly151 (98% occupancy) of NS3 and its carbonyl backbone makes two hydrogen bonds one each with Gly153 (70% occupancy) and Tyr161 (99% occupancy) of NS3 (Figure 4B; Supplementary Figure S9B). Interestingly, the P3 K moved toward Asp83^*^ of NS2B and is making a weak ionic interaction with it (46% occupancy). However, as F84^*^ of NS2B rotated away from P3 K, it could not interact with F84^*^. These interaction profiles indicate that although YKRKST makes stable interactions with the residues of non-prime sites (S1–S3), it failed to interact strongly with the prime site residues (S1' and S2' sites). This likely is the cause of its lower stability than that of YRRRST.

### Effect of the Protein Dynamics

**Figure 5:**
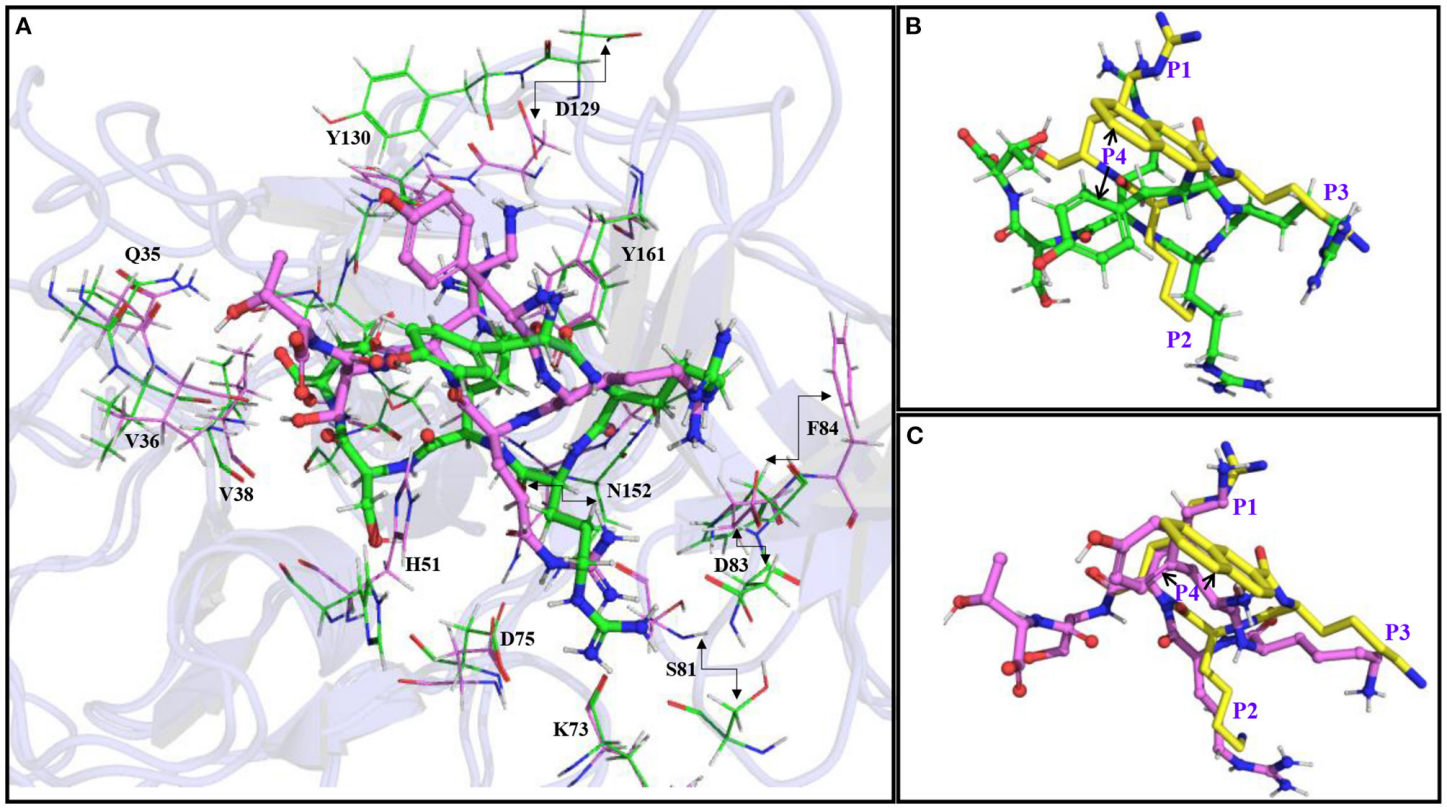
**(A)** Superimposition of the average simulated structure of the YRRRST-protease complex (carbon atoms are shown in green) onto the average simulated structure of the YKRKST-protease complex (carbon atoms are shown in violet). **(B)** Superimpositions of the average simulated structures of the YRRRST-protease complex and **(C)** YKRKST-protease complex onto the peptidomimetic–protease complex containing KKR (PDB ID 5ZMQ) (carbon atoms in yellow). The movements of some residues during the simulations are indicated by arrows. The P1–P4 residues of the inhibitors are labeled in **(B,C)**.

The superimposition of the average structures of YRRRST and YKRKST bound to NS2B–NS3 protease of ZIKV indicates that the loop regions of the protease containing residues from S1, S2, and S3 sites move significantly during the simulations. In the case of the YRRRST-protease complex, the movement of the S1 site away from the P1 R is significant compared to that of the YKRKST-protease complex (Figures 4, 5; Supplementary Figure S9). Due to this reason, the P1 R of YRRRST is weakly bound to the S1 site (Figure 5A). Although the loop containing K73, D75, etc. of NS2B that partly constitutes the S2 site does not move much, the loop containing S81^*^, D83^*^, F84^*^, etc. of NS2B moves significantly in the YRRRST-protease complex. As was observed earlier (35) and is evident from Figure 5A, His51 is quite flexible and can adopt different conformations as per the ligand orientation (Figure 5A). This structural reorientation in the YRRRST-protease complex created a wide and deep S1 pocket (Supplementary Figure S12). Similarly, a wide pocket is also found at the prime site (Supplementary Figure S12).

Interestingly, except for the C-terminal primed residues (P1' and P2'), non-primed residues (P1–P3) of YRRRST and YKRKST adopted identical conformations. The conformation of P4 Y is slightly different in these two peptides as it is exposed to solvent and therefore, enjoys conformational flexibility. If we compare the conformation of YRRRST and YKRKST with the X-ray conformation of a peptidomimetic inhibitor-containing KKR (PDB ID 5ZMQ) (30), it is clear that P1, P2, and P3 residues of all of these inhibitors adopt an identical conformation (Figures 5B,C). However, the structural similarity of YKRKST with the peptidomimetic inhibitor is more rigorous (Figure 5C). These results indicate that the hexapeptides would act as potent inhibitors of protease activities. It also indicates that the preference of basic residues, such as K, R, and K at P1, P2, and P3 positions are not necessary if the peptide inhibitor is extended at the C-terminal. Nevertheless, the presence of basic residues (K/R) at the P1–P3 positions is required to get optimal inhibitory activities. Due to structural similarities between ZIKV, WNV, and DENV, it is expected that these inhibitors may act as pan-flavivirus inhibitors.

It should be mentioned that as the protease cleaves the substrate by cleaving the P1-P1' CN bond (Supplementary Figure S11) with the help of Ser135, His51, and Asp75 of NS3, the hexapeptides studied herein may be converted to tetrapeptides (30, 53). We found that in the case of YRRRST, the Ser135 and Gly133 oxyanion hole are moved away from the carbonyl O of the P1 residue and hence cannot make any hydrogen bond with it (Supplementary Figure S11A). This type of inactive conformation of oxyanion hole was reported earlier (54). However, the same oxyanion hole is active in the case of YKRKST as evident by two strong hydrogen bonds made by Gly133 and Ser135 with the carbonyl O of the P1 residue of the YKRKST (Supplementary Figure S11B). Interestingly, as the C (P1 residue)-O (Ser135) bond distance is almost identical (3.4 Å in YKRKST and 3.6 Å ion YRRRST) in these two complexes (Supplementary Figure S11), the Ser135 may get covalently bonded to the P1 carbonyl, thereby eventually leading to the scission of the P1-P1' peptide bond (53). Nevertheless, the results obtained herein highlight the importance of extended inhibitors in fully occupying the substrate-binding site of the protease. As residues beyond S2' site participate in the membrane-binding (36), further extension of the hexapeptide inhibitors may not yield encouraging results. Therefore, it is likely that hexapeptide inhibitors that contain an unnatural backbone may not be cleaved by the protease and hence would be extremely useful in inhibiting protease activities. Further, as in all of the inhibitors studied herein, the N-terminal P4 Y extends toward the C-terminal residue; cyclic inhibitors that connect C- and N-terminals (32, 33) may also be useful in inhibiting the protease activities. Similarly, the use of peptidomimetics or small molecular ligands that can produce equivalent interactions as generated by basic residues and are capable of crossing the hydrophobic lipid bilayers would be highly beneficial.

## Conclusion

The present study revealed that the binding of a tetrapeptide inhibitor to the NS2B–NS3 protease of the ZIKV would create a highly stable complex (ΔG_bind_ = ~ −81 kcal/mol) when the P1, P2, and P3 positions of the inhibitor contain K, R, and K, respectively. As the P4 position is exposed to solvent and acts as an anchoring group to stabilize the peptide in the substrate-binding site, Y at this position would serve the purpose. The presence of consecutive Rs at P1, P2, and P3 positions and Y at the P4 position would create the second most stable peptide-protease complex (ΔG_bind_= ~ −78 kcal/mol). However, the consideration of other combinations of K and R at these positions would also generate potent inhibitors as the ΔG_bind_ of such inhibitor-protease complexes lie between ~-59 and −74 kcal/mol. The C-terminal extension of YRRR and YKRK by including P1' S and P2' T would generate even more stable complexes with ΔG_bind_ = ~-107 kcal/mol and ~ −97 kcal/mol, respectively. These hexapeptides would behave as better substrate-competitive inhibitors than those of the tetrapeptide inhibitors. Thus, this study has indicated that there is enormous potential to develop potent inhibitors of the ZIKV protease by modifying the C-terminal prime residues with unnatural backbones or unnatural amino acids.

## Data Availability Statement

The original contributions presented in the study are included in the article/Supplementary Materials, further inquiries can be directed to the corresponding author.

## Author Contributions

NRJ conceived the research, analyzed the results, and wrote the paper. SP performed simulations. NRJ and SP approved the paper. Both authors contributed to the article and approved the submitted version.

## Conflict of Interest

The authors declare that the research was conducted in the absence of any commercial or financial relationships that could be construed as a potential conflict of interest.

## Publisher's Note

All claims expressed in this article are solely those of the authors and do not necessarily represent those of their affiliated organizations, or those of the publisher, the editors and the reviewers. Any product that may be evaluated in this article, or claim that may be made by its manufacturer, is not guaranteed or endorsed by the publisher.
