# Supplementary material for: C-Terminal Extended Hexapeptides as Potent Inhibitors of the NS2B-NS3 Protease of the ZIKA Virus
Source: Front Med (Lausanne). 2022 Jul 6;9:921060. doi: 10.3389/fmed.2022.921060 (PMC9306491; doi:10.3389/fmed.2022.921060)
Supplement: Supplementary file 1 [file Data_Sheet_1.PDF]

Supplementary Material

C-terminal Extended Hexapeptides as Potent Inhibitors of the NS2B-NS3 Protease of the ZIKA Virus

S. Pant[a] and N.R. Jena[b]*

[a]Department of Pharmacoinformatics, National Institute of Pharmaceutical Education and Research, Kolkata-700054, India

[b]Discipline of Natural Sciences, Indian Institute of Information Technology, Design and Manufacturing, Dumna Airport Road, Jabalpur-482005, India

Corresponding Author's Email Address: nrjena@iiitdmj.ac.in

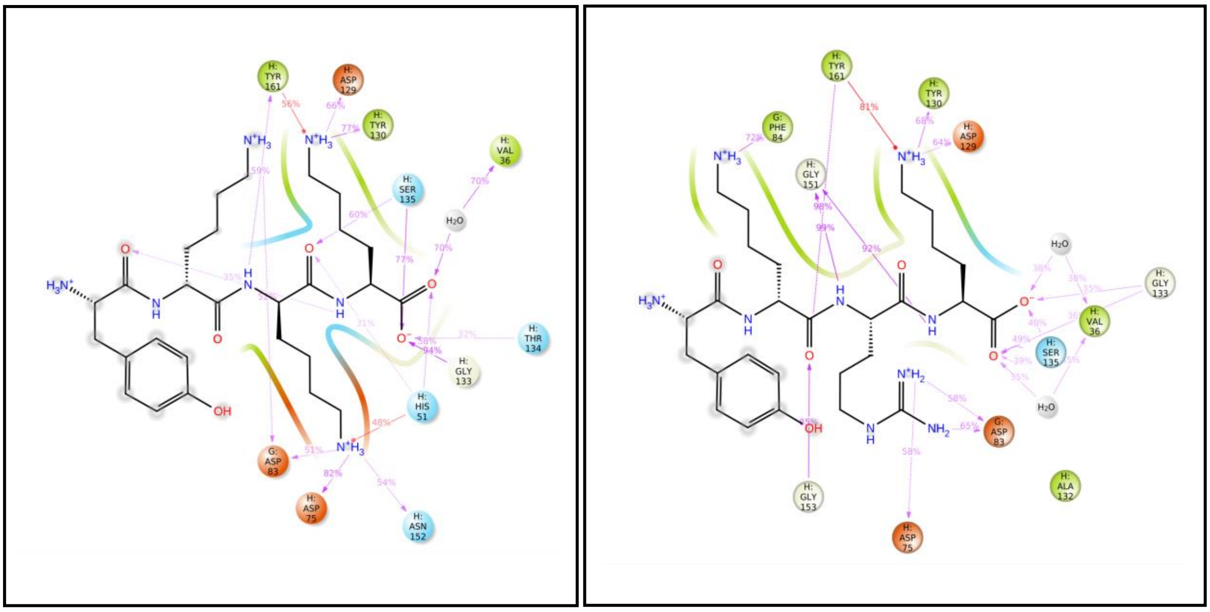

(a) YKKK [-59.16±6.69]   **(b) YKRK** [-80.52±6.84]

Charged (negative) Hydrophobic Water Salt bridge
Glycine Polar Pi-cation Solvent exposure

Fig. S1: The interaction diagrams showing percentage occupations of different interactions involving (a) YKKK and (b) YKRK and the protease that lasted for more than 30% of total simulation time. The MM/GBSA relative binding free energies (kcal/mol) are shown in brackets. Colour codes used to illustrate different types of interactions are also shown.

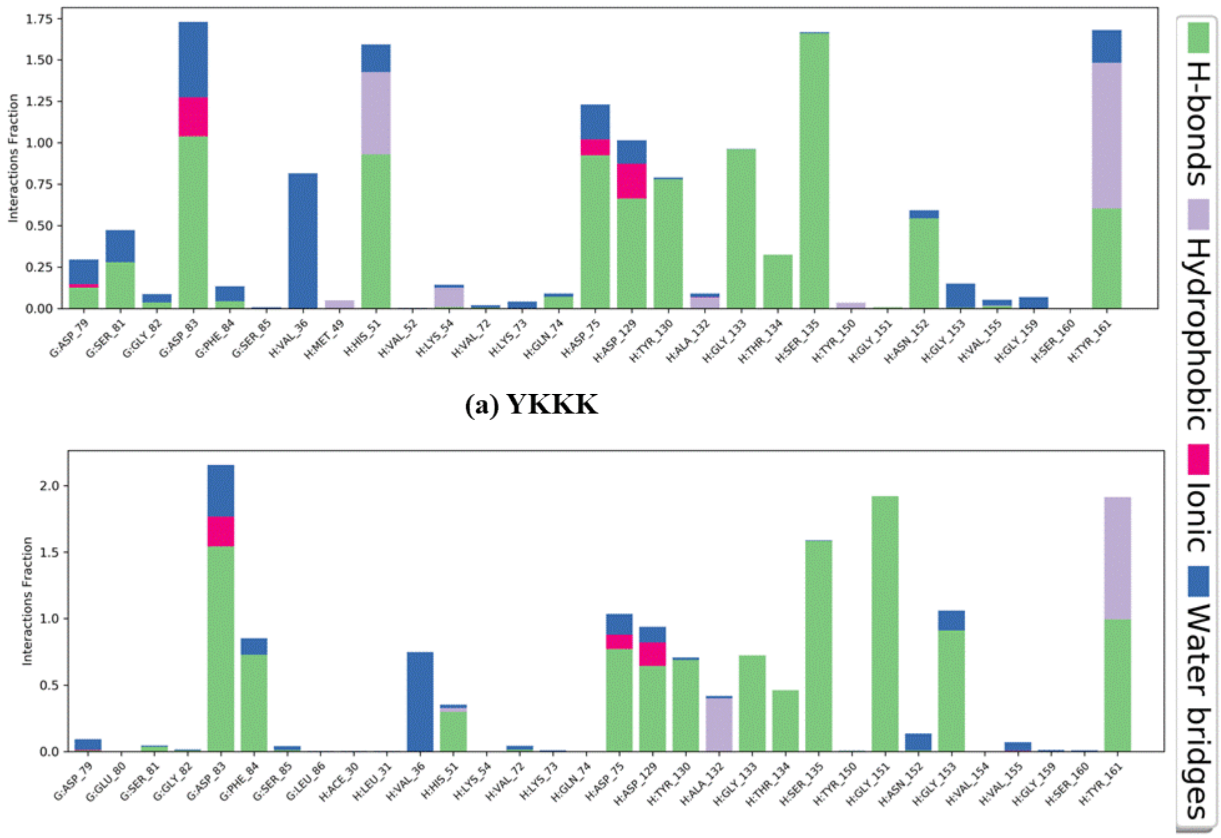

(a) YKKK

(b) YKRK

Fig. S2: The stacked bar charts showing the occupancies of different peptide-protein interactions in (a) YKKK-protease and (b) YKRK-protease complexes obtained throughout the simulations. The interactions fraction over 1.0 indicates that some protein residue may make multiple contacts of the same subtype with the peptide. Colour codes used to illustrate different types of interactions are also indicated.

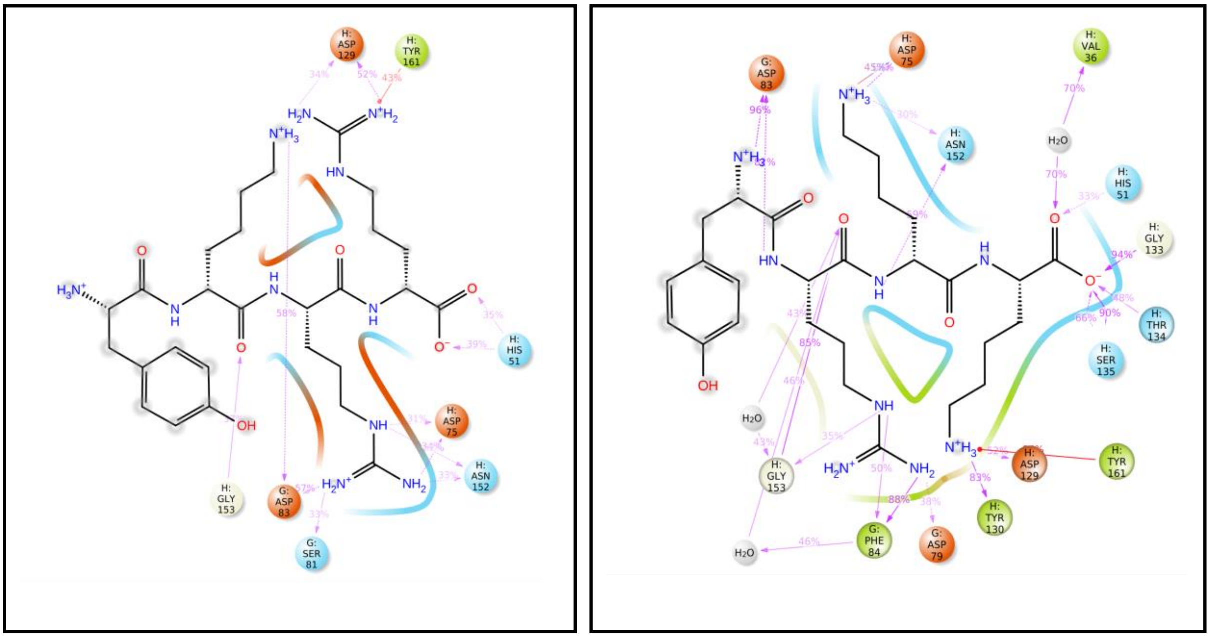

(a) YKRR [-62.47±8.25]   **(b) YRKK** [-72.87±7.25]

Charged (negative) Hydrophobic Water Salt bridge
Glycine Polar Pi-cation Solvent exposure

Fig. S3: The interaction diagrams showing percentage occupations of different interactions involving (a) YKRR and (b) YRKK and the protease that lasted for more than 30% of the total simulation time. The MM/GBSA relative binding free energies (kcal/mol) are shown in brackets. Colour codes used to illustrate different types of interactions are also shown.

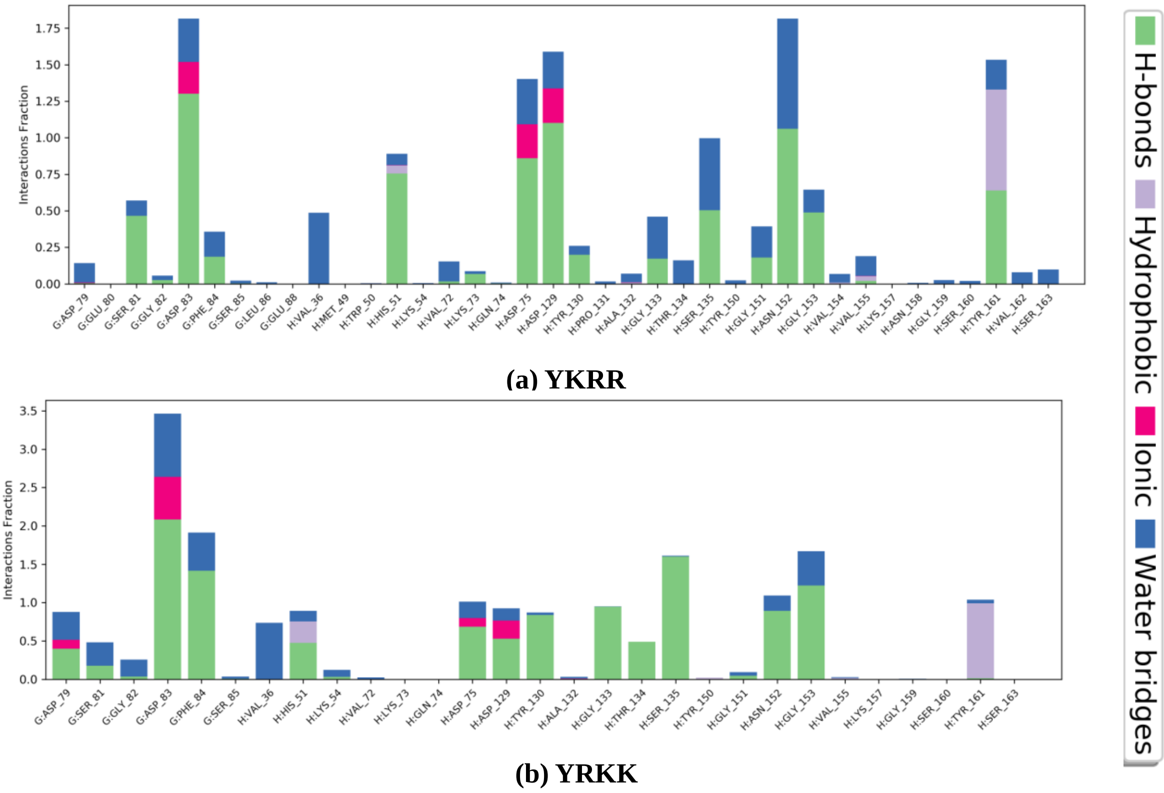

(b) YRKK

Fig. S4: The stacked bar charts showing the occupancy of different peptide-protein interactions in (a) YKRR-protease and (b) YRKK-protease complexes obtained throughout the simulations. The interactions fraction over 1.0 indicates that some protein residue may make multiple contacts of the same subtype with the peptide. Colour codes used to illustrate different types of interactions are also indicated.

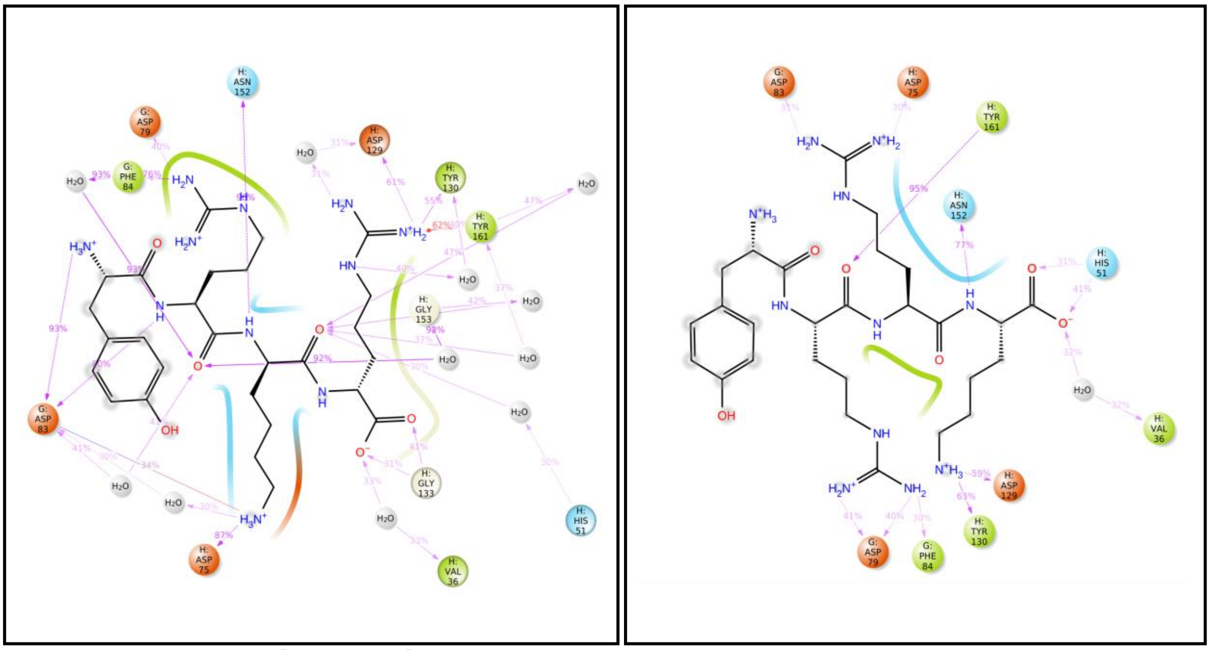

(a) YRKR [-56.33±7.92] **(b) YRRK** [-74.02±9.08]

Charged (negative) Hydrophobic Water Salt bridge
Glycine Polar Pi-cation Solvent exposure

Fig. S5: The interaction diagrams showing percentage occupations of different interactions involving (a) YRKR and (b) YRRK and the protease that lasted more than 30% of total simulation time. The MM/GBSA relative binding free energies (kcal/mol) are shown in brackets. Colour codes used to illustrate different types of interactions are also shown.

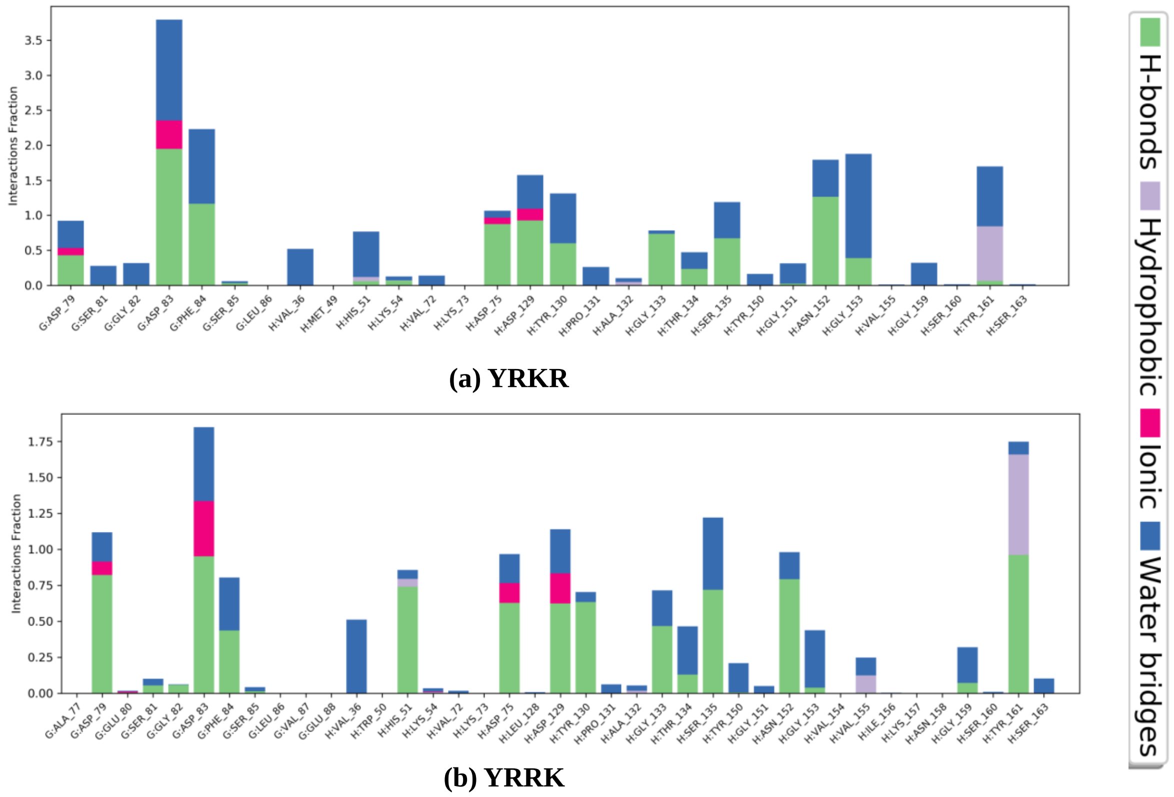

(a) YRKR

(b) YRRK

Fig. S6: The stacked bar charts showing the occupancies of different peptide-protein interactions in (a) YRKR-protease and (b) YRRK-protease complexes obtained throughout the simulations. The interactions fraction over 1.0 indicates that some protein residue may make multiple contacts of the same subtype with the peptide. Colour codes used to illustrate different types of interactions are also indicated.

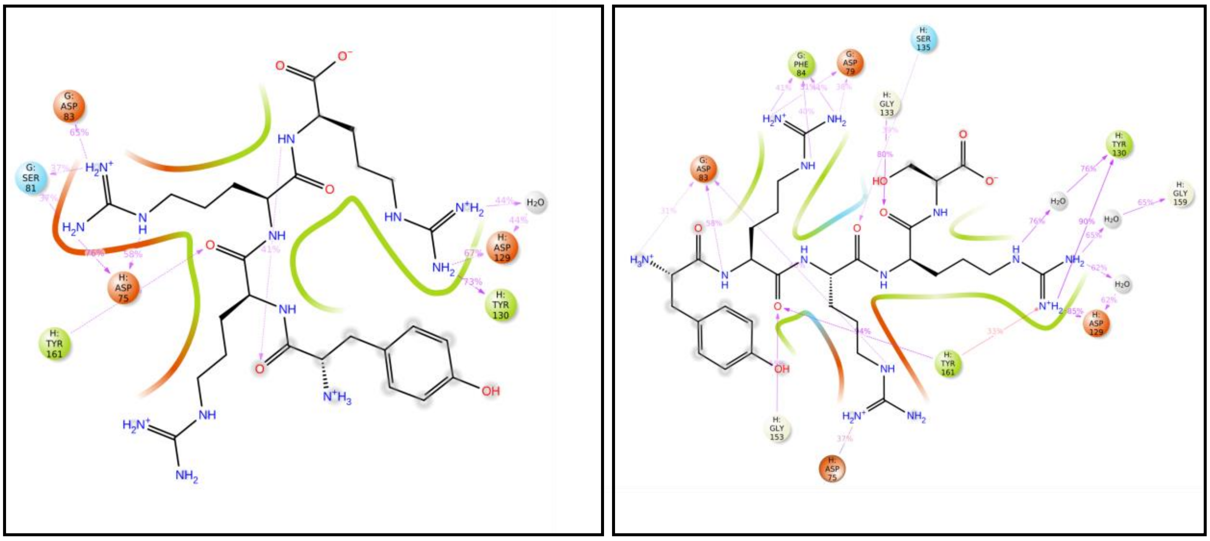

(a) **YRRR** [-77.94±6.90]

(b) **YRRRS** [-84.84±8.46]

Charged (negative) Hydrophobic Water Salt bridge
Glycine Polar Pi-cation Solvent exposure

Fig. S7: The interaction diagrams showing percentage occupations of different interactions involving (a) YRRR and (b) YRRRS and the protease that lasted more than 30% of total simulation time. The MM/GBSA relative binding free energies (kcal/mol) are shown in brackets. Colour codes used to illustrate different types of interactions are also shown.

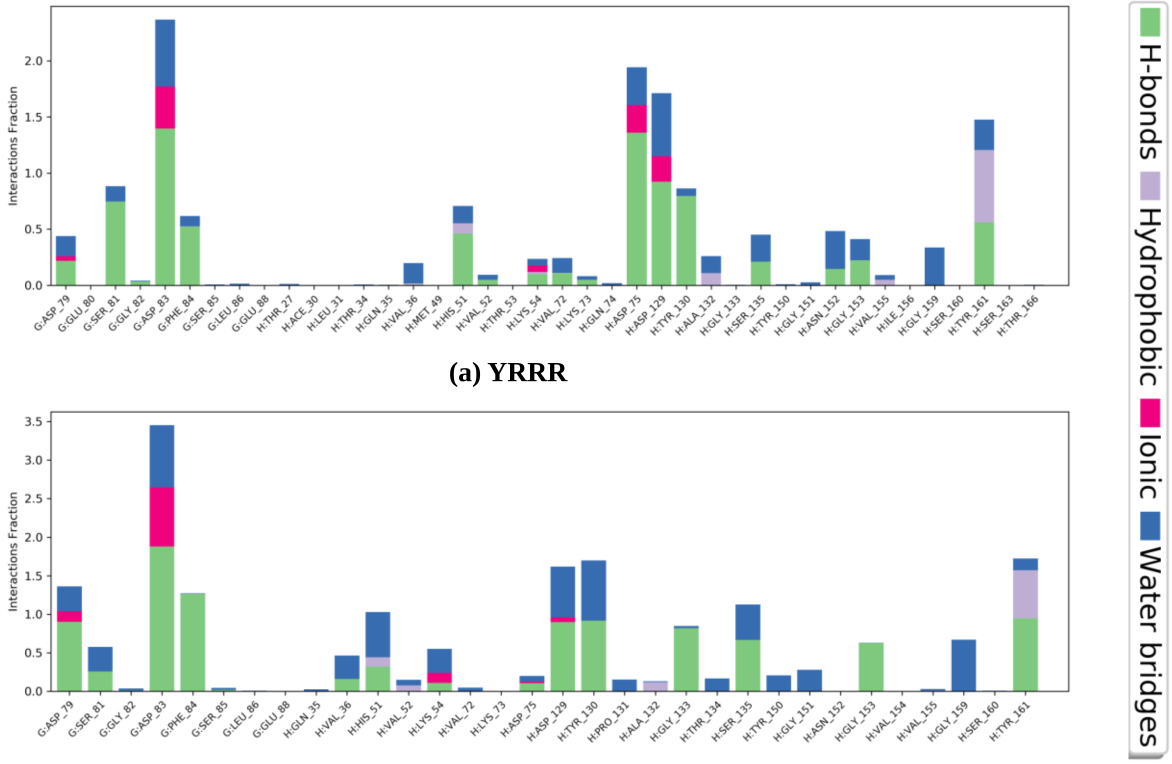

(a) YRRR

(b) YRRRS

Fig. S8: The stacked bar charts showing the occupancies of different peptide-protein interactions in (a) YRRR-protease and (b) YRRRS-protease complexes obtained throughout the simulations. The interactions fraction over 1.0 indicates that some protein residue may make multiple contacts of the same subtype with the peptide. Colour codes used to illustrate different types of interactions are also indicated.

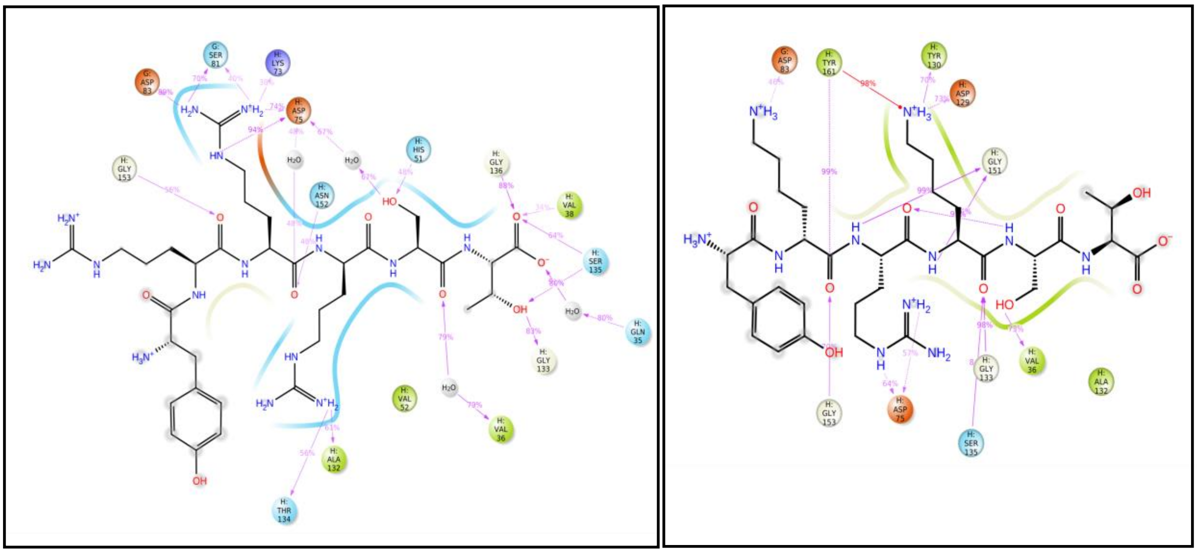

(a) **YRRRST** [-107.49±10.40]

(b) **YKRKST** [-96.84±9.21]

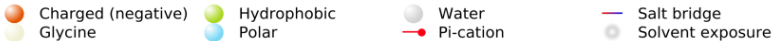

Fig. S9: The interaction diagrams showing percentage occupations of different interactions involving (a) YRRRST and (b) YKRKST and the protease that lasted more than 30% of the total simulation time. The MM/GBSA relative binding free energies (kcal/mol) are shown in brackets. Colour codes used to illustrate different types of interactions are also shown.

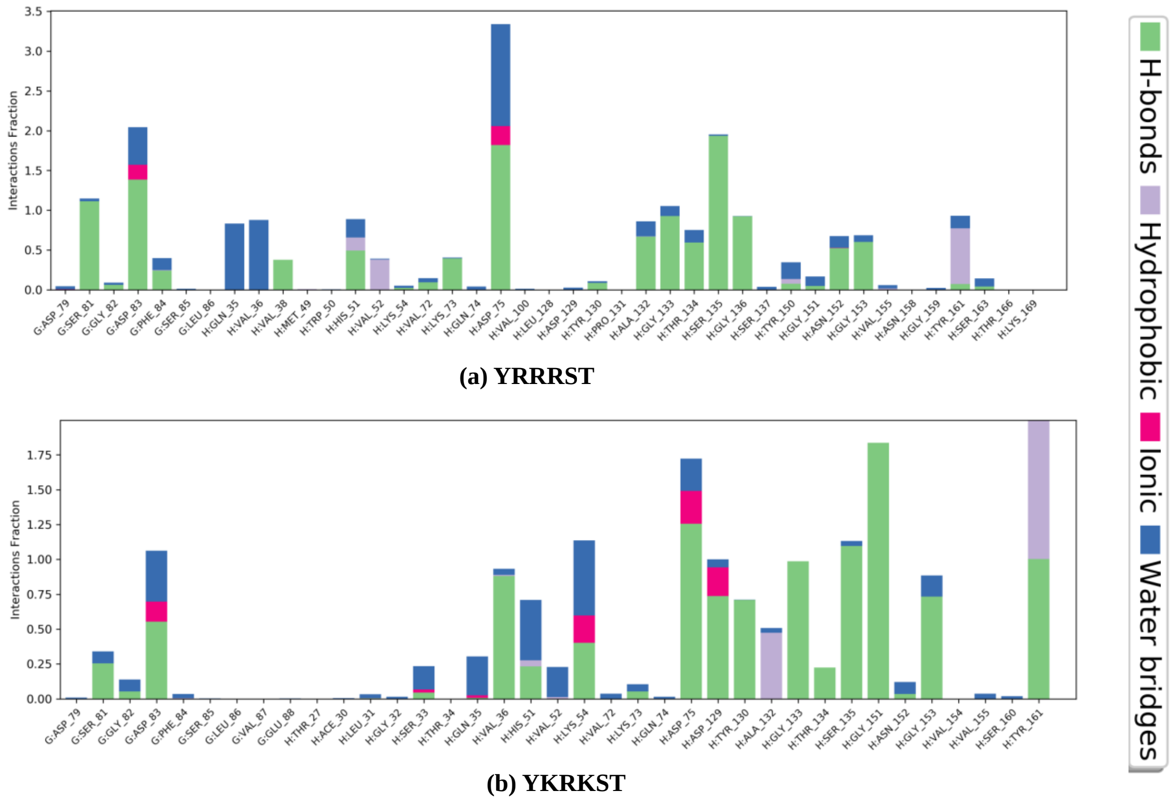

(a) YRRRST

(b) YKRKST

Fig. S10: The stacked bar charts showing the occupancies of different peptide-protein interactions in (a) YRRRST-protease and (b) YKRKST-protease complexes obtained throughout the simulations. The interactions fraction over 1.0 indicates that some protein residue may make multiple contacts of the same subtype with the peptide. Colour codes used to illustrate different types of interactions are also indicated.

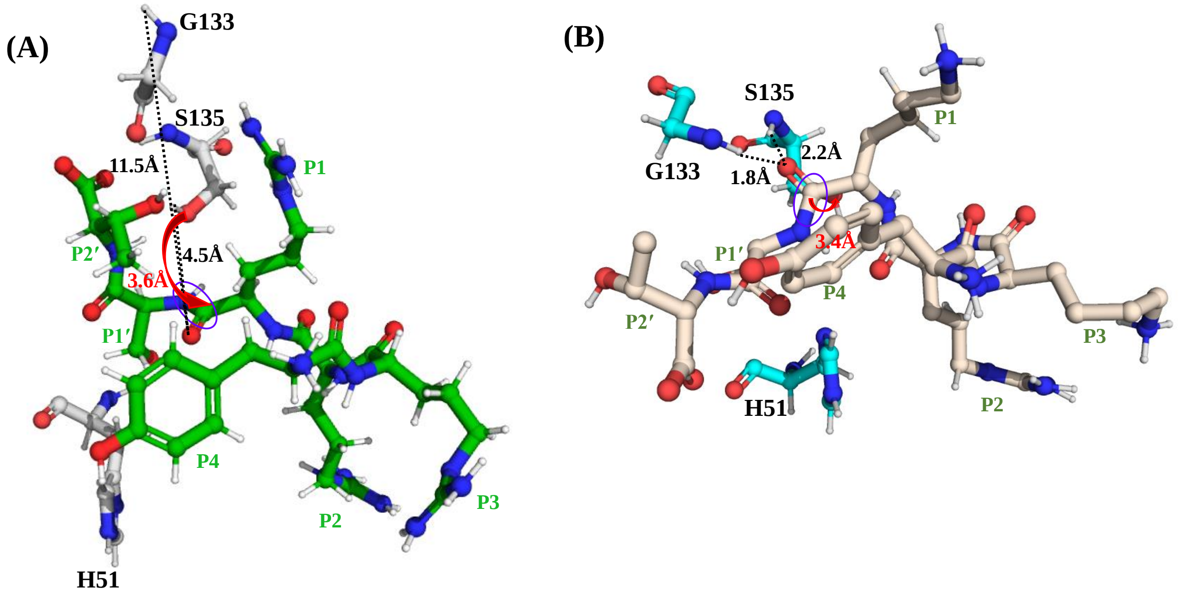

Fig. S11: Placements of some of the catalytically important residues of the protease near (A) YRRRST and (B) YKRKST peptides in their respective average simulated structure. The scissile CN peptide bond between P1 and P1′ residues is marked in a blue circle. The oxyanion hole distances and the C (P1)-O (Ser135) bond distance are shown in Å.

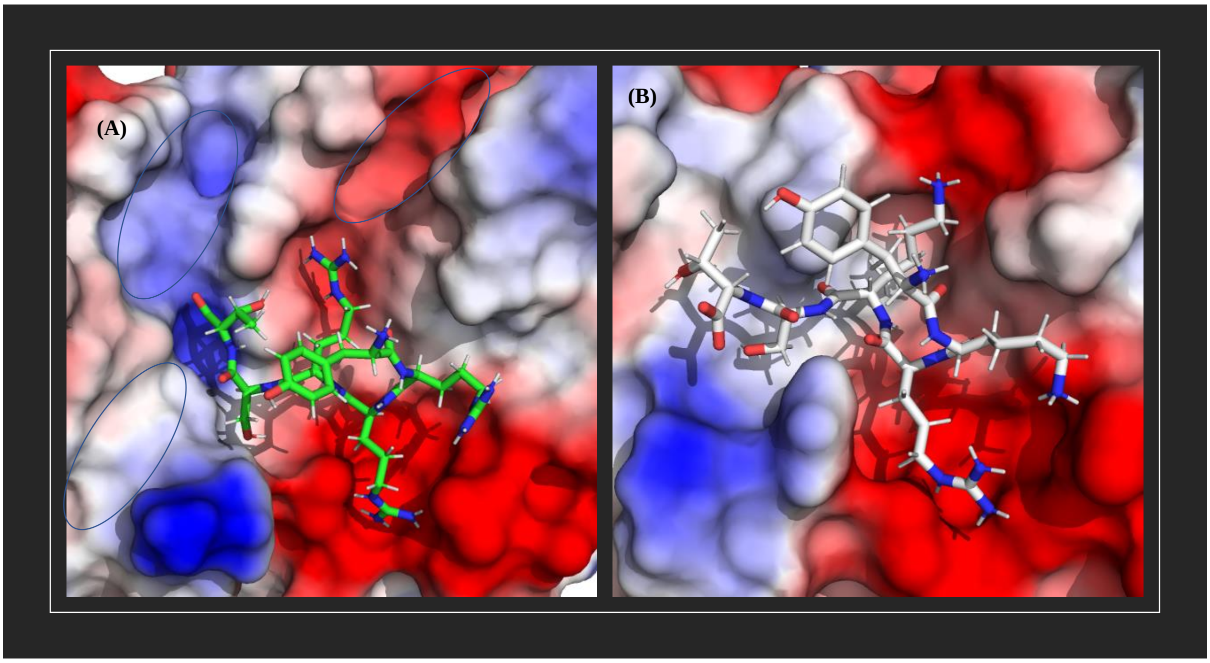

Fig. S12: The electrostatic surface of the (A) YRRRST-protease and (B) YKRKST-protease complexes. The deep and wide pockets created in (A) after ligand binding are marked by circles.
